# An Investigation of Factors Related to Food Intake Ability and Swallowing Difficulty After Surgery for Thoracic Esophageal Cancer

**DOI:** 10.1007/s00455-019-10010-3

**Published:** 2019-04-29

**Authors:** Taichi Mafune, Shinya Mikami, Takehito Otsubo, Osamu Saji, Tsunehisa Matsushita, Takeharu Enomoto, Futaba Maki, Shinobu Tochimoto

**Affiliations:** 1 Department of Gastrointestinal and General Surgery, St. Marianna University School of Medicine, 2-16-1 Sugao, Miyamae-ku, Kanagawa, 216-8511 Japan; 2 Department of Neurology, St. Marianna University School of Medicine, Kanagawa, Japan; 3 Department of Rehabilitation Medicine, St. Marianna University School of Medicine, Kanagawa, Japan

**Keywords:** Postoperative swallowing difficulty, Recurrent laryngeal nerve paralysis, Esophageal cancer surgery, Deglutition, Deglutition disorders

## Abstract

Swallowing difficulty is among the major complications that can occur after surgery for thoracic esophageal cancer. Recurrent laryngeal nerve paralysis (RLNP) has been considered the most significant cause of a postoperative swallowing difficulty, but association between the two has not been adequately explained. We investigated the relation between postoperative RLNP and swallowing difficulty by means of video fluoroscopy. Our study included 32 patients who underwent subtotal esophagectomy for thoracic esophageal cancer at St. Marianna University School of Medicine between April 2014 and March 2017. We evaluated patients’ age and sex, disease stage, preoperative presence of a swallowing difficulty, nutritional status, extent and duration of surgery, blood loss volume, and postoperative presence of RLNP and/or hoarseness. Patients were divided into two groups according to whether oral food intake was possible when video fluoroscopy was performed on postoperative day (POD) 7, and we analyzed the associated factors. Postoperative RLNP occurred in 21 patients (65.6%); hoarseness occurred in 19 (59.4%). Eleven patients (34.4%) suffered swallowing difficulty that prevented food intake. No significant association was found between postoperative swallowing difficulty and postoperative RLNP or hoarseness, but a significant relation was found between the prognostic nutritional index and intraoperative lymph node dissection. Multivariable analysis revealed a significant relation between postoperative swallowing difficulty and only one factor: cervical lymph node dissection (*P* = 0.0075). There appears to be no relation between RLNP pursuant to esophageal cancer surgery and swallowing difficulty that prevents oral food intake.

## Introduction

In Japan, dissection of the lymph nodes around the recurrent laryngeal nerve (RLN) during surgery for thoracic esophageal cancer is considered important. This is a radical form of treatment, and the current standard lymphadenectomy procedure is three-field dissection, which includes cervical nodes around the RLN [1, 2]. The invasiveness of surgery involving the RLN is substantial, and complications following such surgery are more common than complications following other gastrointestinal surgeries [3]. Postoperative swallowing difficulty in particular is well known [4, 5], and reports to date have indicated that recurrent laryngeal nerve paralysis (RLNP) is closely related to such swallowing difficulty [6–9]. The clinical signs of the two disorders, according to the Clavien–Dindo (C–D) classification system, which grades postoperative complications, and the Japan Clinical Oncology Group (JCOG) classification system, which also grades postoperative complications, are quite similar [10, 11]. According to at least one report, postoperative swallowing difficulty cannot be explained by RLNP alone, so there is still room for discussion regarding the etiology of swallowing difficulty in patients who have undergone surgery for esophageal cancer [12].

Video fluoroscopy (VF) is a dynamic diagnostic modality that has been adopted for evaluating swallowing function after surgery for esophageal cancer [13, 14]. We used VF to evaluate swallowing function after surgery for thoracic esophageal cancer, and in addition to investigating the incidence of such swallowing difficulty, we used various intraoperative and preoperative variables, including the presence of RLNP, to gain insight into the etiology of postoperative swallowing difficulty.

## Methods

### Patients

This retrospective clinical study was approved by the St. Marianna University School of Medicine institutional review board (Approval No. 2598). Included in the study were 32 patients with thoracic esophageal cancer treated by subtotal esophagectomy that included anastomosis or cervical lymph node dissection but no extraesophageal surgery. All were treated at St. Marianna University Hospital between April 2014 and March 2017, and consent to use their anonymized data for research purposes had been obtained from all individual participants included in the study.

### Surgical Procedures

The thoracic esophageal cancer was treated by thoracoscopic or laparoscopic-assisted subtotal esophagectomy, which are the standard surgical procedures used at our hospital. Patients undergoing the surgery were placed in a left lateral recumbent position, and the six ports were placed on the right side of the body. Thoracic lymph node dissection was performed in all patients in accordance with the extent of dissection stipulated by the Japan Esophageal Society. Nodes 105, 106rec, 107, 108, 109, 110, and 111 were dissected, and the esophagus was then dissected [15]. Particularly focused bilateral dissection of area 106rec nodes, which surround the RLN, was also performed in all patients. Once the thoracic procedure was completed, the patient was placed in the supine position, and the abdominal procedure was performed. The cervical procedure was performed simultaneously by an otolaryngology surgeon. The lymph node dissection included areas 1, 2, 3a, 7, 9, and 20 and was followed by construction of a narrow gastric tube. The cervical procedure was performed on patients with cancer of the upper or middle thoracic esophagus and superior mediastinal lymphadenopathy. If bilateral dissection of area 101 lymph nodes was required, both subclavian arches were transected; the infrahyoid muscles were not dissected but rather left intact. If additional dissection of the area 104 nodes was required, the sternocleidomastoid muscle over the area of dissection was first transected. Upon completion of the dissection, a gastric tube was passed upward via the posterior mediastinal route, and cervical esophagogastric anastomosis was performed. The sternocleidomastoid muscle was repaired by suturing. In patients in whom cervical lymph node dissection was not performed, the left subclavian arch was transected, the infrahyoid muscles were left intact, traction was applied to the esophagus, and anastomosis was performed.

### Evaluation of RLNP

Laryngoscopy was performed at bedside on postoperative day (POD) 1, and RLNP was diagnosed when vocal cord paralysis (VCP) was detected upon this examination and regardless of whether the paralysis was unilateral or bilateral. In addition, patients were examined physically on POD 7, and hoarseness detected at that time was taken as indirect evidence of RLNP.

### Evaluation of Swallowing Function by Means of VF

Contrast VF was performed preoperatively and on POD 7 for evaluation of patients’ swallowing function. VF evaluation involves analysis of a lateral view obtained with the patient in a seated position and is performed as a joint exercise by a speech therapist, neurologist, and gastroenterologist. The contrast material used to define the esophageal morphology was prepared by diluting 35 mL of a nonionic low-osmolar contrast medium, iopamidol, in 40 mL of water, and then mixing the solution with 1 g of thickener. For both VF examinations, each patient swallowed 5 mL of this contrast material, VF was performed, and the images were examined for an impaired pharyngeal phase reflex, pharyngeal residue, laryngeal invasion, or aspiration. Swallowing difficulty was diagnosed if any of the aforementioned was observed. The patients’ ability to ingest food was examined on VF images obtained on POD 7. An inability to ingest food was diagnosed if one or more of the following criteria were met: evidence of aspiration, evidence of laryngeal invasion preventing rapid clearance by coughing, or evidence of a large amount of pharyngeal residue that could not be cleared by additional swallowing, despite absence of evidence of aspiration or laryngeal invasion [14].

### Evaluation of Factors Contributing to Postoperative Swallowing Difficulty

The following preoperative variables were obtained from patients’ clinical records. Patients’ sex, age, existence of a swallowing disorder, stage of the esophageal cancer, T-factor (depth of invasion), N-factor (lymph node metastasis), and nutritional status (according to the Onodera prognostic nutritional index [PNI] [16] and prealbumin [PA] concentration). The disease stage, T-factor, and N-factor corresponded to the TNM classification [17]. The following intraoperative variables were noted: duration of surgery, duration of the cervical procedure, blood loss volume, and performance of cervical lymph node dissection. The following postoperative variables were noted: presence of RLNP during laryngoscopy on POD 1 and hoarseness on POD 7.

### Statistical Analyses

Study variables were compared between patients with an intact ability to ingest food and those in whom this ability was absent, and differences were analyzed by means of univariable logistic regression analysis or Fisher’s exact test. Association between clinical factors detected by VF on POD7 and RLNP was determined on the basis of odds ratios. Variables for which a *P* value < 0.20 was obtained by univariable analysis were entered into a multinominal logistic regression analysis in which *P* < 0.05 was considered statistically significant. JMP software (version 12; SAS Institute, Cary, NC, USA) was used for all statistical analyses.

## Results

Characteristics of the 32 study patients are shown in Table 1.

**Table 1:** Characteristics of the 32 study patients treated for thoracic esophageal cancer

Sex	
Male	27 (84.3)
Female	5 (15.7)
Age, median (range) years	70.5 (48–81)
T factor	
T1	10 (31.2)
T2	3 (9.4)
T3	19 (59.4)
N factor	
N0	12 (37.5)
N1	12 (37.5)
N2	8 (25)
Pathological stage	
IA	8 (25.0)
IB	1 (3.1)
IIA	3 (9.4)
IIB	3 (9.4)
IIIA	12 (37.5)
IIIB	5 (15.6)
PA, median (range)	25 (13–34)
PNI, median (range)	46 (37.6–54.4)
Preoperative swallowing disorder	
Present	11 (34.4)
Not present	21 (65.6)
Operation time (min), median (range)	466.5 (320–650)
Duration of cervical procedures (min), median (range)	230.5 (150–355)
Blood loss (mL), median (range)	227.5 (40–921)
Cervical lymph node dissection	
Yes	19 (59.4)
No	13 (40.6)
Postoperative laryngoscopy findings	
RLNP present	21 (65.6)
RLNP not present	11 (34.4)
Postoperative hoarseness	
Present	19 (59.4)
Not present	13 (40.6)

Number (and percentage) of patients are shown unless otherwise indicated

*PA* prealbmin concentration, *PNI* Onodera prognostic nutritional index, *RLNP* recurrent laryngeal nerve palsy diagnosed on the basis of vocal cord paralysis (VCP)

### Incidences of Postoperative RLNP and Hoarseness

RLNP was observed during postoperative laryngoscopy in 21 (65.6%) of the 32 study patients. Nineteen (59.4%) of the 32 patients complained of postoperative hoarseness, and the hoarseness in these patients was confirmed upon clinical examination (Table 1).

### VF Findings

Some type of abnormality was evident during postoperative VF in 24 (75%) of the 32 patients, as shown in Table 2. Swallowing difficulty that was sufficiently severe to cause an inability to ingest food was diagnosed in 11 (34.4%) patients.

**Table 2:** Incidence and details of VF-detected pharyngeal-phase swallowing disorders

VF finding	Present	Not present
Some type of swallowing disorder	24 (75%)	8 (25%)
Impaired pharyngeal reflexes	8 (25%)	24 (75%)
Pharyngeal residue	24 (75%)	8 (25%)
Laryngeal invasion	11 (34.4%)	21 (65.6%)
Aspiration	5 (15.6%)	27 (84.4%)
Swallowing disorder with AFIA^a^	11 (34.4%)	21 (65.6%)

*VF* videofluoroscopy, *AFIA* absence of food intake ability

^a^AFIA despite observation of a large amount of pharyngeal residue, laryngeal invasion, or aspiration

### Relation Between RLNP and Swallowing Difficulty Diagnosed on POD 7 Based on an Inability to Ingest Food

Nine (42.8%) of the 21 patients in whom RLNP was diagnosed on the basis of VCP were unable to ingest food postoperatively, with the remaining 12 (57.2%) able to ingest food (Table 3). Two (18.2%) of the total 11 patients without RLNP were unable to ingest food. There was, however, no significant relation between a swallowing difficulty diagnosed on POD 7 on the basis of an inability to ingest food and the presence of RLNP (*P* = 0.25) (Table 4).

**Table 3:** RLNP and hoarseness in relation to postoperative food intake ability

	RLNP present	RLNP not present	Total
Postoperative IFIA	12	9	21
Postoperative AFIA	9	2	11
Total	21	11	32

	Hoarseness present	Hoarseness not present	Total
Postoperative IFIA	11	10	21
Postoperative AFIA	8	3	11
Total	19	13	32

Number of patients is shown

*RLNP* recurrent laryngeal nerve palsy diagnosed on the basis of vocal cord paralysis (VCP), *IFIA* intact food intake ability, *AFIA* absence of food intake ability

**Table 4:** Results of univariable analysis of preoperative and intraoperative IFIA and AFIA in relation to study variables

Variable	Postoperative IFIA (*n*=21)	Postoperative AFIA (*n*=11)	OR (95% CI) *P* value
Male/female sex	18/3	9/2	1.333 (0.188–9.475) 1.00
Age (median years, interquartile range)	70 (59–75.5)	71 (65–76)	0.985 (0.905–1.064) 0.70
T factor T1/T2/T3	7/3/11	3/0/8	0.49
N factor N0/N1/N2	9/8/4	3/4/4	0.57
Stage IA/IB/IIA/IIB/IIIA/IIIB	6/1/2/3/7/2/	2/0/1/0/5/3	0.66
PA (median, interquartile range)	27 (22.5–30.5)	23 (17–29)	1.113 (0.975–1.296) 0.11
PNI (median, interquartile range)	47.4 (44.9–49.5)	44 (41.9–46)	1.300 (1.047–1.718) 0.016
Preoperative swallowing disorder			
Yes	6	5	
No	15	6	0.48 (0.105–2.191) 0.44
Operation time (median min, interquartile range)	480 (435–540)	436 (423–536)	1.002 (0.993–1.013) 0.62
Duration of cervical procedures (median min, interquartile range)	235 (204–269)	218 (192–253)	1.011 (0.996–1.030) 0.15
Blood loss (median mL, interquartile range)	228 (154–309)	223 (106–524)	0.999 (0.995–1.003) 0.57
Cervical lymph node dissection			
Yes	9	10	
No	12	1	0.075 (0.008–0.697) 0.011
Postoperative laryngoscopy finding			
RLNP present	12	9	
RLNP not present	9	2	0.296 (0.051–1.721) 0.25
Postoperative hoarseness			
Yes	11	8	
No	10	3	0.413 (0.085–2.001) 0.45

*OR* odds ratio*, PA* prealbmin concentration, *PNI* Onodera prognostic nutritional index, *RLNP* recurrent laryngeal nerve palsy diagnosed on the basis of vocal cord paralysis (VCP)

Similarly, as shown in Table 3, 8 (42.1%) of the 19 patients with hoarseness were unable to ingest food, and 11 (57.9%) of the 19 were able to ingest food. Of the 13 patients without hoarseness, 3 (23%) were unable to ingest food. No significant relation was found between swallowing difficulty diagnosed on POD 7 based on the inability to ingest food and the presence of hoarseness (*P* = 0.45) (Table 4).

### Relation Between Postoperative Swallowing Difficulty and Preoperative and Intraoperative Variables Assessed

When we analyzed study variables in relation to swallowing difficulty including absence of the ability to take in food orally (Table 3), we found the PNI (*P* = 0.015) and cervical lymph node dissection (*P* = 0.010) to be significant. When we entered items shown to be significant by univariable analysis into the multinominal logistic regression model (Table 4), we found cervical lymph node dissection to be significantly related to absence of the food intake ability (*P* = 0.0075) (Table 5).

**Table 5:** Results of multivariable analysis of factors related to absence of food intake ability

Factor	OR	95% CI	*P* value
PA	1.053	0.853–1.334	0.632
PNI	1.197	0.858–1.782	0.295
Duration of cervical procedures	1.015	0.994–1.043	0.160
Cervical lymph node dissection	18.707	2.008–532.960	0.0075

*OR* odds ratio, *CI* confidence interval, *PA* prealbmin concentration, *PNI* Onodera prognostic nutritional index

### Relation Between RLNP and VF Findings

No significant association was found between the presence of RLNP and any of the factors examined by VF, i.e., between RLNP and an impaired laryngeal reflex, pharyngeal residue, laryngeal invasion, or aspiration (Table 6).

**Table 6:** Relations between VF finding on postoperative day 7 and RNLP

VF finding	RLNP present (*n *= 21)	RLNP not present (*n *= 11)	OR (95% CI)
Impaired pharyngeal reflexes			0.556 (0.092–1.064) *P*=0.68
Present	15	9	
Not present	6	2	
Pharyngeal residue			0.833 (0.158–4.401)
Present	16	8	*P*=1.00
Not present	5	3	
Laryngeal invasion			0.609 (0.124–2.996) *P*=0.70
Present	8	3	
Not present	13	8	
Aspiration			*P*=0.14
Present	5	0	
Not present	16	11	

*VF* video fluoroscopy, *RLNP* recurrent laryngeal nerve paralysis, *OR* odds ratio, *CI* confidence

## Discussion

Our principal objective in investigating swallowing difficulty after surgery for thoracic esophageal cancer was to determine whether food intake is possible during the early postoperative period. In addition to clarifying the incidence of swallowing difficulty, we investigated the effects of RLNP and other preoperative and intraoperative factors on absence of the food intake ability during this period. We also examined association between RLNP, and factors examined by VF during the pharyngeal phase of swallowing.

As noted above, laryngoscopic examination revealed RLNP in 65.6% of our patients. This incidence of RLNP may seem high in comparison with incidences previously reported, but the previously reported diagnoses of RNLP were based strictly on clinical symptoms such as hoarseness, and the time between surgery and diagnosis differed from the time between the two among our patients. Therefore, comparison between our data and data previously reported is difficult. For our study, RNLP was defined as either unilateral or bilateral VCP. In one reported study of the incidence of post-esophagectomy RNLP, the disorder was diagnosed by laryngoscopic examination, but RNLP was defined only as bilateral VCP, and the RNLP was documented in about 50% of patients 2 weeks after the surgery. According to reports to date of diagnosis of RLNP by laryngoscopic examination, the incidence of RLNP following surgery for esophageal cancer ranges from 36% to 75%. The incidence among our patients falls within this range. RNLP has been reported to improve by more than 50% during postoperative follow-up. We believe, then, that RNLP is present in about 65% of patients on POD 1, but that the percentage decreases gradually with time. We also believe that RNLP can be present in patients without clinical symptoms.

Meanwhile, 34.4% of our patients were without an inability to take food orally, and this differed from the 65.6% of patients who exhibited RLNP. Our data suggest that postoperative swallowing difficulty may be independent of the presence of RLNP. The incidence of postoperative swallowing difficulty did not differ between patients with and without RLNP, despite the fact that RLNP has been considered a major factor in postoperative swallowing difficulty, according to studies to date. Surprisingly, in this study, we found no relation between RLNP and the factors identified by VF, laryngeal invasion and aspiration. We know that various nerves and muscles work together to effect airway protection and swallowing during the pharyngeal phase. The glossopharyngeal nerve induces the pharyngeal phase reflex, and the airway is then closed by the vocal cords, the false vocal cords, and the epiglottis. This is followed by simultaneous elevation of the hyoid and opening of the esophageal orifice, after which the food bolus is moved into the esophagus by the squeezing motion of the pharyngeal constrictors. With the exception of the cricothyroid muscle, the laryngeal muscles are supplied by the RLN. Accordingly, vocal cord closure is impaired when RLNP occurs [14]. Thus, ineffective closure of the airway increases the risk of aspiration. However, airway protection is not established by vocal cord closure alone. It is due also to the position and movement of the false vocal cords and to the movement of the epiglottis [18]. This means that aspiration in patients is not necessarily due to the RLNP. Basic research performed in animal models has indicated that the larynx is mostly closed even after transection of one or both RLNs, and this does not affect swallowing [19]. Thus, we believe that it is important to consider the fact that postoperative swallowing difficulty cannot be explained by RLNP alone.

Our study yielded two points of major clinical significance. The first is the clear relation between aspiration caused by RLNP and postoperative swallowing difficulty, and this relation is reflected in the C–D and JCOG classification systems, which are generally used to grade postoperative complications. However, it is not possible to correctly evaluate complications, even when these tools are used [10, 11]. The C–D system considers treatment of complications to be the principal objective of classification. The C–D system does not specify RLNP as a specific item but instead includes it under the respiratory section. The JCOG system, which is based on the C–D system, considers either aspiration or RLNP as fulfilling a single criterion. We did not observe any relation between a swallowing difficulty and RLNP in our study patients, so this simple criterion might not allow for accurate evaluation of complications. The C–D system represents complications according to their severity, which is based on the degree of treatment needed for them. Under this system, RLNP is classified according to the degree of treatment needed for aspiration pneumonia resulting from the RLNP. However, when RLNP and swallowing difficulty can be directly evaluated by VF and video esophagography, it might be possible to prevent aspiration in advance. It is even possible that the VF evaluation prevented aspiration in some of our patients, which would have influenced our data. By extension, therefore, we believe postoperative evaluation by means of both VF and video esophagography is necessary for accurate evaluation of RLNP and swallowing dysfunction.

The second point of clinical significance is the dissociation between swallowing disorders and symptoms that suggest RLNP. Physical findings that suggest the presence of aspiration or RLNP, such as hoarseness or coughing, are commonly used in clinical settings to determine when to initiate food intake. However, our study did not show any relation between hoarseness and a postoperative swallowing difficulty. In terms of the dissociation between swallowing function and clinical symptoms, a previous study has included many patients with silent aspiration who did not present with hoarseness, indicating that it may not be possible to predict swallowing difficulty on the basis of physical findings alone [7]. We believe that the results of our study support this conclusion.

We investigated the relation not only between postoperative swallowing difficulty and the presence of RLNP but also between postoperative swallowing difficulty and various preoperative and intraoperative variables. Among these items, we noted a significant association for the item “cervical lymph node dissection.” Moreover, as mentioned above, we found no significant relation between swallowing difficulty and RLN injury. Cervical esophageal anastomosis is a standard procedure at our hospital, so all of our study patients underwent a cervical procedure on the left side during surgery for esophageal cancer. Furthermore, adding cervical lymph node dissection expanded the extent of the cervical procedure bilaterally. Among recently reported studies in which swallowing difficulty has been evaluated by means of VF after surgery for esophageal cancer, two have shown inadequate laryngeal elevation in patients with a postoperative swallowing difficulty [18, 20]. Results of these studies suggested that swallowing difficulty might be caused by inflammation and scarring that result from the cervical procedure. Results of one study are particularly interesting: that swallowing function appeared to be preserved to a similar extent between patients in whom cervical lymph node dissection was not performed and patients in whom the infrahyoid muscles were transected bilaterally to facilitate laryngeal elevation after cervical lymph node dissection [12]. In considering these results comprehensively, we believe that inflammation and scarring of the infrahyoid muscles, as well as incomplete relaxation due to muscle injury, are possible causes of postoperative swallowing difficulty. We believe that these factors also contributed to our study results. The fact that the incidence of swallowing difficulty increased to a greater extent when cervical lymph node dissection, rather than cervical anastomosis, was performed suggests that the risk of these disorders increases as the extent of the cervical procedure is expanded.

Our study was not without limitations. The first was the small number of patients included. This is important with respect to the analyses of T-factors, N-factors, and disease stages. It was also not possible for us to incorporate tumor location, and the extent of cervical lymph node dissection into our analysis because the number of patients in each category was small. Going forward, we will need to accumulate additional cases. The second limitation was that it was not possible to examine the relation between details of the RLNP and swallowing difficulty. In this study, RLNP was diagnosed regardless of whether the VCP was unilateral or bilateral. Factors such as unilateral vs. bilateral VCP, the degree of paralysis, degree of vocal cord atrophy, and reflex time were not documented laryngoscopically. In addition, laryngoscopy was performed only in the acute phase on POD 1, and it was difficult for patients to move to the examination room or ingest food. Therefore, we were limited to briefly observing the presence or absence of RLNP at bedside. However, because there have been many studies evaluating swallowing difficulty in relation to unilateral VCP, we believe that RLNP as we defined it was a valid study endpoint [18, 21]. The third limitation was that the study was a single center study. A standardized surgical method was used, making it easy for us to perform a comparison to invest factors related to swallowing dysfunction in patients who have undergone surgery, including lymphadenectomy, for thoracic esophageal cancer. However, there are reports of differences in the incidence of swallowing dysfunction based on the anastomotic method used [6], so we hope, going forward, to evaluate postoperative swallowing function by means of VF jointly with other institutions that make use of different surgical techniques. The fourth limitation was the fact that assessment of food intake ability by means of VF is somewhat subjective. Three criteria for absence of food intake ability were established and included in our study, and these were agreed upon based on the opinions of the speech therapist, neurologist, and gastroenterologist. VF has been considered a subjective means of evaluation [14]; in recent years, there have been some objective VF-based assessments that have incorporated measurable variables, such as laryngeal elevation (reported as a percentage) and pharyngeal transit time [18, 22]. We therefore believe that assessment of postoperative food intake will become more objective if these methods are pursued and reference values are established.

In conclusion, postoperative swallowing difficulty after surgery for thoracic esophageal cancer is affected very little by the presence of RLNP but greatly by inclusion of cervical lymph node dissection in the operative procedure. Thus, it is difficult to determine when to initiate postoperative food intake simply on the basis of the presence of RLNP and its associated symptoms, as we have done to date, and we believe that VF is a powerful tool that can be used for dynamic assessment. The C–D system can, to some degree, be relied upon for classification of RLNP and swallowing difficulty. We believe that the standards need to be reestablished to take VF and video esophagography findings into consideration.
